# Zn(II) Induces Fibril Formation and Antifungal Activity in Shepherin I, An Antimicrobial Peptide from *Capsella bursa-pastoris*

**DOI:** 10.1021/acs.inorgchem.3c03409

**Published:** 2023-11-20

**Authors:** Joanna Wątły, Klaudia Szarszoń, Aleksandra Mikołajczyk, Manuela Grelich-Mucha, Agnieszka Matera-Witkiewicz, Joanna Olesiak-Bańska, Magdalena Rowińska-Żyrek

**Affiliations:** † Faculty of Chemistry, University of Wrocław, F. Joliot-Curie 14, 50-383 Wrocław, Poland; ‡ Screening of Biological Activity Assays and Collection of Biological Material Laboratory, Wrocław Medical University Biobank, Faculty of Pharmacy, Wrocław Medical University, 50-556 Wrocław, Poland; § Faculty of Chemistry, Wrocław University of Science and Technology, Wyb, Wyspiańskiego 27, 50-370 Wrocław, Poland

## Abstract

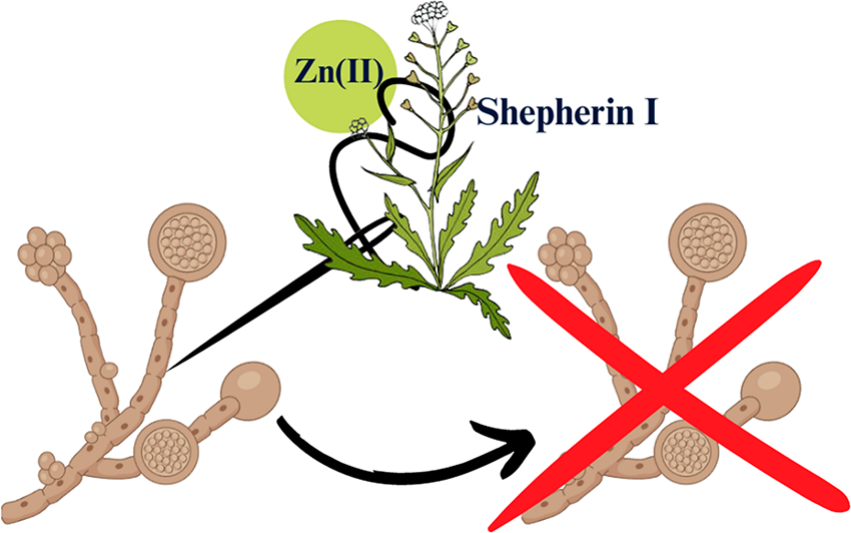

Shepherin I is a glycine- and histidine-rich antimicrobial peptide from the root of a shepherd’s purse, whose antimicrobial activity was suggested to be enhanced by the presence of Zn(II) ions. We describe Zn(II) and Cu(II) complexes of this peptide, aiming to understand the correlation between their metal binding mode, structure, morphology, and biological activity. We observe a logical sequence of phenomena, each of which is the result of the previous one: (i) Zn(II) coordinates to shepherin I, (ii) causes a structural change, which, in turn, (iii) results in fibril formation. Eventually, this chain of structural changes has a (iv) biological consequence: The shepherin I–Zn(II) fibrils are highly antifungal. What is of particular interest, both fibril formation and strong anticandidal activity are only observed for the shepherin I–Zn(II) complex, linking its structural rearrangement that occurs after metal binding with its morphology and biological activity.

## Abstract 2

The coordination of Zn(II) (but not that of Cu(II)) triggers the antifungal properties of shepherin I, a glycine- and histidine-rich antimicrobial peptide from the root of shepherd’s purse. Zn(II) binding causes a structural change, which triggers fibril formation. Fibrils are most likely relevant for establishing a new mechanism of fungal cell wall or membrane disruption.

## Introduction

Antimicrobial peptides (AMPs) are small, usually polycationic peptides, isolated from natural sources, showing antibacterial, antiviral, antifungal, and even anticancer activity. As a result, AMPs represent promising alternative agents to overcome increasing antibiotic resistance problems.^1^ They are also known as host defense peptides due to being essential components of immune response of multicellular organisms.^2^ So far, more than 3500 AMPs have been reported and are diverse in biological source, activity, structure, and mechanism of action.^3^

Generally, AMPs have been separated into several groups based on their (i) biological source; (ii) peptide sequence; (iii) secondary structure; (iv) covalent bonding pattern; (v) biosynthesis route; (vi) molecular targets; (vii) antibacterial target; and (viii) high abundance of specific amino acids.^4^

It is not uncommon for antimicrobial peptides to be rich in one particular amino acid; the antimicrobial peptide database^3^ contains 117 specific “Xaa-rich” antimicrobial peptides; the most abundant ones are Pro-rich (56), Gly-rich (31), Arg-rich (20), and His-rich (15). Shepherin I is classified as both Gly-rich and His-rich.

The structural arrangement of AMPs is essential to understand their interaction mechanisms with biological targets. AMPs have three main modes of action: interaction with membrane phospholipids leading to their disruption,^5,6^ intracellular targeting in order to inhibit synthesis of nucleic acids, enzymes, and other crucial proteins,^7^ and also by less popular types such as molecular electroporation and sinking raft mechanisms^8^ (The first one uses electric potential to form a pore and disruption of the membrane; this mechanism is possible due to charged properties of peptides. The second one suggests that AMPs accumulate on the membrane and modify its shape; the aggregation of these peptides presses into the bilayer and sinking inside via transitional pore transporting them to the other side of the membrane.).

What is of particular interest in this work is the fact that both Zn(II)^9,10^ and Cu(II)^1,11^ are able to change the structure and increase the antimicrobial activity of antimicrobial peptides. Both metal ions are essential micronutrients and, being quite abundant in the human body (most commonly bound to other biomolecules), have to be considered an important AMP enhancement factor. Zinc(II), being bound to ca. 10% of all proteins, is 1 order of magnitude more abundant in the human body than copper, and is normally relatively nontoxic compared to other metals, e.g., copper.^12,13^

There are two metal-related activities of AMPs: (i) process named “nutritional immunity” which is based on binding metal ions essential for their virulence and life^13^ and (ii) when metal ions enhance antimicrobial activity of AMPs by changing their structure or/and charge.^14^ Examples of AMPs for which antimicrobial activity is metal-related are clavanins, pramlintide, calcitermin, or semenogelins.^15−18^

We focus on shepherin I, isolated from the roots of the plant *Capsella bursa-pastoris* and its antimicrobial activity in complexes with metal ions. We show how metal ions, such as Cu(II) and Zn(II) influence on the thermodynamic and structural properties of shepherin I, and we suggest the possible mechanisms of their antimicrobial action.

*C. bursa-pastoris*, also known as shepherd’s purse, is one of the most abundant flowering plants worldwide.^19^ Despite the fact that it is a plant that is well-known as a widespread and difficult to eradicate weed that grows massively in fields, pastures, and gardens, is has been used in medicine since ancient times.^20^ It has a huge pool of extremely valuable compounds such as flavonoids, sterols, vitamins, and metal ions necessary for health and well-being. Studies have shown its antihemorrhagic, antibacterial, anti-inflammatory, and anticancer properties.^21^ Alkaloids and flavonoids of *C. bursa-pastoris* show high antibiotic potencies and broad antimicrobial spectra. The plant’s antimicrobial properties were for a long time attributed mainly to sulforaphane, isothiocyanate compound, active against Gram-positive bacterium *Bacillus anthracis* and vancomycin-resistant *Enterococci* strains,^20^ until two novel peptides were isolated from this plant, which show activity against Gram-negative bacteria and fungi: shepherin I (Shep I) and shepherin II (Shep II).^22^

**Figure 1:**
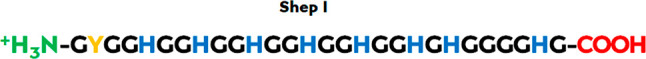
Amino acid sequence of shepherin I isolated from *C. bursa-pastoris*.

The sequence of shepherin I (Shep I) is quite uncommon and, for the same reason, easy to describe: It consists of 19 Gly, 8 His and 1 Tyr residue, or, in other words, almost 67.9% of its sequence are Gly residues and 28.6% are His residues (Figure 1). This peptide is also characterized by seven repeats of tripeptide GGH (Gly-Gly-His)–moreover six of them are adjacent.^23^

Far-UV CD spectra of shepherin I showed no helical structure in 50% trifluoroethanol,^22^ but β-pleated sheet structures were observed in 60% TFE and 20 mM SDS.^23^

Glycine has no side chain and is too flexible to participate in the hydrogen bonds required for secondary structure such as α-helices and β-sheets, however, in some cases specific cross-strand pairing with aromatic residues can improve the stabilization of Gly residue in β sheets.^24^ Nevertheless, literature data indicate that Gly (and also Pro) residues are favored at several positions of some β-turn types.^25,26^

Previous studies show that the glycine-rich shepherin and its analogues are effective against Gram-negative bacteria including *Escherichia coli*, *Pseudomonas putida*, *Pseudomonas syringae*, and *Serratia sp.*, and against yeast phase-fungi, like *Candida albicans*, *Cryptococcus neoformans*, and *Saccharomyces cerevisiae*. Moreover, the addition of 10 μM ZnCl_2_ improved the activity of Shep I (and its C-terminal amidated analogue) against some strains of *C. albicans* (including fluconazole-resistant strain).^23^

We focus on understanding the thermodynamic and structural properties of Shep I, and its Zn(II) and Cu(II) complexes, in order to understand the correlation with their antimicrobial properties and propose a potential mechanism of action.

## Experimental Section

### Materials and Methods

H_2_N-GYGGHGGHGGHGGHGGHGGHGHGGGGHG-COOH (shepherin I, Shep I) peptide (certified purity of 98%) was purchased from KareBay Biochem and used without further purification. Cu(II) and Zn(II) perchlorides were extra-pure products (Sigma-Aldrich); concentration of their stock solutions was determined by ICP–MS. The carbonate-free stock solution of 0.1 M NaOH (Sigma-Aldrich) was potentiometrically standardized with potassium hydrogen phthalate (Sigma-Aldrich). All of the samples were prepared with freshly doubly distilled water. The ionic strength (*I*) was adjusted to 0.1 M by the addition of NaClO_4_ (Sigma-Aldrich). All of the samples were weighted out using analytical scale Sartorius R200D.

### Mass Spectrometry

High-resolution mass spectra were obtained on Bruker Apex FT-ICR and Shimadzu q-TOF LCMS 9030 spectrometers. Spectrometers were used for measurements on Cu(II) and Zn(II) complexes in the ranges of positive and negative values. The instrumental parameters were as follows: scan range *m*/*z* 150–2000; dry gas nitrogen; temperature 170 °C; capillary voltage 4500 V; ion energy 5 eV. The Cu(II) and Zn(II) complexes [(metal/ligand stoichiometry of 1:1) [ligand]_tot_ = 100 μM] were prepared in a 50:50 MeOH/H_2_O mixture at pH 6. The samples were infused at a flow rate of 3 μL/min. Data were processed by application of the Bruker Compass DataAnalysis 4.0 program and the ACD/Spectrus Processor.

### Potentiometry

Stability constants for proton and Cu(II) and Zn(II) complexes were calculated from titration curves carried out over the pH range of 2.50–12.00 at *T* = 298 K in a total volume of 3 mL. The pH-metric titrations were performed in 0.004 M HClO_4_ and 0.1 M NaClO_4_ ionic strength (both ligands are soluble in pure water solution). The potentiometric titrations were performed with a Metrohm 809 Titrando pH-meter titrator provided with Mettler-Toledo glass-body, micro combination pH electrode. The glass cell was equipped with a magnetic stirring system, a micro buret delivery tube, and an inlet–outlet tube for high-purity grade argon in order to maintain an inert atmosphere. Solutions were titrated with 0.1 M carbonate-free NaOH. The electrode were calibrated daily for hydrogen ion concentration by titrating HClO_4_ with alkaline solution in the same experimental conditions as above. Purities and the exact concentrations of the ligand solutions were determined by the Gran method.^27^ The ligand concentration was 0.5 mM. The metal-to-ligand ratio was 0.9:1 for metal complexes. The standard potential and the slope of the electrode couple were computed by means of the Glee program.^28^ HYPERQUAD2006 program was used for the stability constant calculations.^29^ The constants for hydrolysis of Cu(II) and Zn(II) ions were taken from literature.^30,31^ The speciation and competition diagrams were computed with the HySS program^32^ and drawn in OriginPro 2016 program.

### Spectroscopy

The absorption spectra in the UV–vis region were recorded at 298 K on a Varian Cary300 Bio spectrophotometer in a 10 mm path length quartz cell. The spectral range was 200–800 nm. Circular dichroism (CD) spectroscopy experiments were performed on a Jasco V-750 spectropolarimeter at 298 K in a 10 mm quartz cell. The spectral range was 190–800 nm. Direct CD measurements (Θ) were converted to mean residue molar ellipticity (Δε) using the Jasco Spectra Manager

Far-UV circular dichroism (CD) spectra were recorded with a JASCO V-715 CD spectropolarimeter at the temperature of 293 K in 0.1 and 0.2 mm path length quartz cells. Every sample contained a peptide at a concentration of 0.3–0.4 mM and 0.9:1 of metal to ligand molar ratio. The samples were prepared in 4 mM HClO_4_ and 0.1 M NaClO_4_ ionic strength. In addition, the peptide titration by using of different concentrations of sodium dodecyl sulfate (SDS; [SDS] = from 10 to 200 mM) was measured. Far-UV CD spectra were recorded from 180 to 250 nm for ligands and complexes at selected pH. Appropriate amounts of NaOH or HClO_4_ solutions were added to change pH values.

### Antimicrobial Activity Assay of Peptide and Peptide-Metal Ion Complex System

Antimicrobial activity was performed by using the broth microdilution method with spectrophotometric measurements. Peptide and peptide-metal ion systems were analyzed. Reference strains from the ATCC collection (*E. coli* 25922, *Staphylococcus aureus* 43300, *Klebsiella pneumoniae* 700603, *Acinetobacter baumannii* 19606, *Pseudomonas aeruginosa* 27853, *Enterococcus faecalis* 29212, and *C. albicans* 10231) were used. The experimental procedure followed the guidelines outlined in ISO standards 20776–1:2019^33^ and 16256:2012,^34^ along with a modified Richard’s method.^35−37^

Stock peptide solution was prepared in distilled sterile water at four times concentration higher than the final one. Equimolar concentrations of Cu(II) and Zn(II) ions were added to the peptide. Serial dilutions of the peptide/peptide-metal ion solutions were prepared in 96-well microplates, covering a concentration range of 0.5– 1256 μg/mL. After 24 h incubation at 37 °C for bacteria or 25 °C for fungus, the bacterial and fungal suspensions were prepared to achieve a final inoculum density of 5 × 10^q5^ CFU/mL (for bacteria) and 0.5–2.5 × 10^5^ CFU/mL (for fungus). A positive controls (TSB + strain) and negative controls (TSB) were also performed. Additionally, a solubility control for each peptide and peptide-metal ion system was also taken into account. To validate the assay, antibacterial/antifungal agents such as levofloxacin (*A. baumannii* 0.5 μg/mL, *E. faecalis* 4 μg/mL, *P. aeruginosa* 1 μg/mL, methicillin-resistant *S. aureus* (*MRSA*, 1 μg/mL), gentamicin (*E. coli* 4 μg/mL, *K. pnemoniae* 4 μg/mL), or amphotericin B (*C. albicans* 1 μg/mL), in accordance with the EUCAST examination, were tested against each strain. Microplates were incubated at 37 ± 1 or 25 ± 1 °C for 24 h on a shaker (500 rpm). After the incubation, spectrophotometric measurements were performed at 580 nm. The minimum inhibitory concentration that inhibits the growth of 50% microorganisms (MIC_50_) was determined by comparing the absorption results of the test samples with the positive control.

Subsequently, 50 μL aliquots of a 1% (m/v) solution of 2,3,5-triphenyltetrazolium chloride (TTC) were added to each well. TTC is a redox indicator used to assess cellular respiration. The colorless TTC is oxidized to pink after reduction due to reactions in the respiratory chain, indicating microbial viability.

By combining the MIC_50_ results with the modified Richard’s method and the TTC indicator, the minimal bactericidal/fungicidal concentration (MBC/MFC) could be determined. MBC/MFC represents the lowest concentration of the antimicrobial agent required to kill the respective microbial strain, as evidenced by the absence of pink due to the lack of enzymatic reduction to red 1,3,5-triphenylformazan (TPF).

### Neutral Red (NR) Cytotoxicity Uptake Assay

NR cytotoxicity uptake investigation was conducted for shepherin I–Zn(II) using human primary renal proximal tubule epithelial cells (RPTEC; ECACC 85011425). Following concentrations were chosen based on results obtained in antimicrobial activity assay: 1, 10, 75, and 125 μM. The experimental procedure followed ISO:10993 guidelines, specifically ISO:10993–5:2009^38^ and ISO/IEC 17025:2005.^39^ The NR assay protocol from Nature Protocol was employed as a standardized approach.^40^

The experiment utilized MEMα supplemented with 10% fetal bovine serum (FBS), 2 mM glutamine, and an appropriate amount of antibiotics (amphotericin B, gentamicin). Stock solution of the Shep I peptide and equimolar concentration of Zn(II) ions were prepared in water and subsequently diluted 100 times in the growth medium. Additionally, solutions of Zn(II) salts were tested to ensure the absence of any potential cytotoxic effects from the metal ions alone. Following the addition of the respective combinations of testing compounds and cells (at a density of 1 × 10^5^ cells/mL) to the wells, the plates were incubated at 37 °C in a 5% CO_2_ environment for 48 and 72 h.

After removing the medium, each well received 100 μL of NR solution (40 μg/mL), followed by a 2 h incubation at 37 °C. Subsequently, the dye was removed, and the wells were rinsed with phosphate-buffered saline (PBS) and allowed to dry. Next, an NR destain solution (consisting of 1% glacial acetic acid, 50% 96% ethanol, and 49% deionized water, v/v) was added to each well. The plates were then shaken (30 min, 500 rpm) to extract NR from the cells and form a homogeneous solution. The absorbance was measured at 540 nm by using a microplate reader. Untreated cells were considered as a negative control, representing 100% potential cellular growth. Additionally, cells incubated with 1 μM staurosporine were used as a positive control for inducing cytotoxicity.

### Atomic Force Microscopy (AFM) Imaging

AFM imaging was performed by using a Dimension V Veeco AFM instrument in the tapping mode with the SSS probe mounted. Morphology of the samples was verified just after their preparation; pH of the samples was set to the value of around 7.40. 30 μL aliquots of the samples were deposited on mica discs, and after a 30 min adsorption period, the samples were rinsed with Milli-Q water and dried. The mean width and height dimensions were calculated based on 50 individual profiles using Gwyddion, an open-source software for scanning probe microscopy data analysis.^41^

## Results and Discussion

### Shepherin I Protonation Constants

Based on a series of potentiometric titrations ten deprotonation constants (p*K*_*a*_) were established for Shep I (GYGGHGGHGGHGGHGGHGGHGHGGGGHG) (Table 1, Figure S1). The determined values are in agreement with those found in the literature for similar systems.^42−48^

**Table 1:** Deprotonation Constants for Shep I Peptide and Stability Constants for Its Complexes with Cu(II) and Zn(II) Ions in an Aqueous Solution of 4 mM HClO_4_ with *I* = 0.1 M NaClO_4_ at 298 K^a^

Shep I				Shep I–Cu(II)			Shep I–Zn(II)		
species	log β_*jk*_^b^	p*K*^b^^,^^c^	residue	species	log β*_jk_*^d^	p*K*_a_^e^	species	log β_*jk*_^e^	p*K*_a_^e^
**[H**_**10**_**L]**^**9+**^	72.20(1)	5.29	His						
**[H**_**9**_**L]**^**8+**^	66.91(2)	5.76	His	[CuH_8_L]^9+^	67.57(5)				
**[H**_**8**_**L]**^**7+**^	61.15(3)	6.04	His	[CuH_7_L]^8+^	63.21(2)	4.36			
**[H**_**7**_**L]**^**6+**^	55.11(5)	6.40	His	[CuH_6_L]^7+^	58.22(2)	4.99	[ZnH_6_L]^7+^	54.09(3)	
**[H**_**6**_**L]**^**5+**^	48.71(6)	6.54	His	[CuH_5_L]^6+^	52.42(2)	5.80	[ZnH_5_L]^6+^	48.35(2)	5.74
**[H**_**5**_**L]**^**4+**^	42.17(4)	6.97	His	[CuH_4_L]^5+^	45.92(2)	6.50	[ZnH_4_L]^5+^	42.04(2)	6.31
[H_4_L]^3+^	35.20(3)	7.21	His	[CuH_3_L]^4+^	38.54(3)	7.38	[ZnH_3_L]^4+^	34.92(2)	7.12
[H_3_L]^2+^	27.99(2)	8.03	His	[CuH_2_L]^3+^	30.74(3)	7.80	[ZnH_2_L]^3+^	26.72(3)	8.20
[H_2_L]^+^	19.96(1)	9.58	NH_2_	[CuHL]^2+^	21.57(4)	9.17	[ZnHL]^2+^	17.27(3)	9.45
[HL]	10.38(1)	10.38	Tyr	[CuL]^+^	11.89(4)	9.68	[ZnL]^+^	7.59(3)	9.68
				[CuH_–1_L]	1.95(3)	9.94			
				[CuH_–3_L]^2–^	–20.00(3)				

a *C*_L_ = 0.5 mM; molar ratio M/L = 0.9:1. The standard deviations are reported in parentheses as uncertainties on the last significant figure.

b Protonation constants are presented as cumulative log β*_jk_* values. β(H*_j_*L*_k_*) = [H*_j_*L*_k_*]/([H]*_j_*[L]*_k_*), in which [L] is the concentration of the fully deprotonated peptide.

c p*K*_a_ = log β(H*_j_*L*_k_*) – log β(H*_j_* – 1L*_k_*).

d Cu(II) and Zn(II) stability constants are presented as cumulative log β*_ijk_* values. L stands for a fully deprotonated peptide ligand that binds Cu(II) and Zn(II) ions. β(M*_i_*H*_j_*L*_k_*) = [M*_i_*H*_j_*L*_k_*]/([M]*_i_*[H]_*j*_[L]_*k*_), where [L] is the concentration of the fully deprotonated peptide.

e p*K*_a_ = log β(M*_i_*H*_j_*L*_k_*) – log β(M*_i_*H*_j_* – 1L*_k_*).

### Shepherin I–Metal Complexes

To investigate the precise stoichiometry, structural and thermodynamic properties of shepherin I–metal complexes set of experimental methods were used: mass spectrometry (MS), potentiometric titrations, and UV–visible and circular dichroism (CD) spectroscopy.

The mass spectra measurements revealed the stoichiometry of the metal complexes, indicating that only equimolar species were present in solution under the tested experimental conditions (Figures S2 and S3). The most intense signals (*m*/*z*) of each systems were identified and assigned to the appropriate species (Table S1). *M/z* values and isotopic distributions align perfectly with the simulated spectra.

#### Shep I–Cu(II) complex

Potentiometric measurements revealed the presence of 11 equimolar complex species for the Shep I–Cu(II) system in the pH range of 2.50–12.00. The complex distribution diagram and stability constants values are shown in Figure S4 and Table 1, respectively. A careful study of the obtained experimental potentiometric and spectroscopic results allows for a detailed thermodynamic and structural characterization of the formed species, showing the number and type of coordinated atoms from the peptides as described in the Supporting Information and summarized in Table S2.

At a physiological pH, Cu(II) is bound to a {2N_im_} donor set. Deprotonations of subsequent imidazole residues, for which a decrease in the p*K*_a_ value is observed (compared to the ligand, indicating the binding of the metal ion (Table 1)), and no significant changes in the UV–vis and CD spectra (Figure S5) may suggest the existence of several complex species in equilibrium, in each of which a maximum of two imidazole nitrogens are bound to copper(II). Such type of binding is referred to as polymorphic binding sites (metal can “move back and forth” along such regions). In case of Shep I, the regularly repeating GGH motif (GGHGGHGGHGGHGGHGGH) is an excellent candidate for this type of metal ion binding.^42,45^

The polymorphic binding mode is an interesting phenomenon used by nature to adjust the outcome of metal coordination to the current physiological requirements. It is observed here for Shep I–Cu(II) at physiological pH, and in numerous other cases, e.g., for Cu(II) complexes with a peptide from snake venom, which contains nine subsequent His residues.^45^

**Figure 2:**
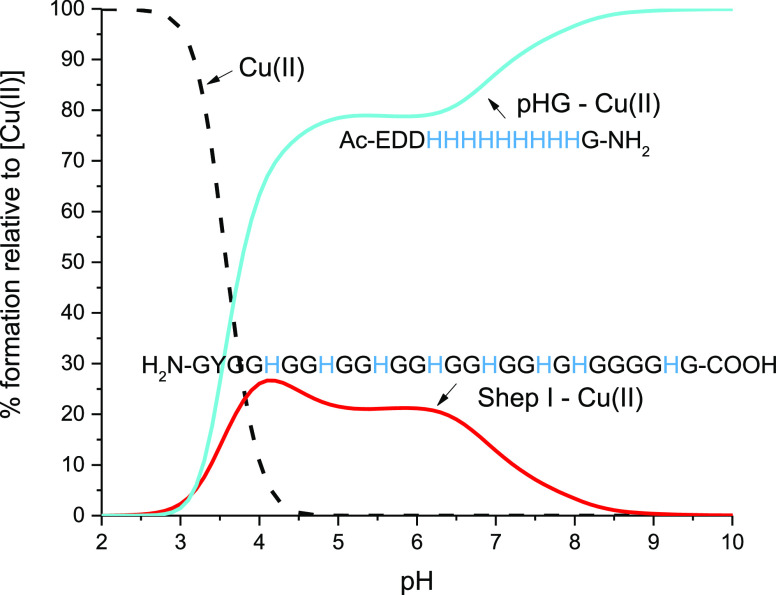
Competition plots for the Cu(II) complexes with Shep I (red) and pHG (cyan; Ac-EDDHHHHHHHHHG-NH_2_) peptides based on potentiometric data (Table 1 and for pHG taken from ref (45)).

We compare the stability of copper complexes of the pHG peptide (from snake venom) to the peptide studied in this work, in which the His residues are separated by Gly repeats, on a competition plot, based on the complexes’ stability constants, which is a hypothetical situation in which equimolar amounts of the three reagents are mixed. The comparison shows that pHG has a higher affinity toward Cu(II) than Shep I (Figure 2). There are at least two reasons for such a big difference: (i) In pHG, Cu(II) is bound to 3 imidazole nitrogens, while in the case of Shep I to two nitrogens and (ii) most interestingly, a significant influence of metal ions on the helical structure formation has been observed in molecular dynamic simulations and also in the circular dichroism spectra for the pHG complex, which may orient the His side chains accordingly, making them more accessible for Cu(II) (which is not the case for Shep I, see the section “Impact of Metal Coordination on Shepherin I Structure”).^45^

#### Shep I–Zn(II) Complex

The stability constants for the Zn(II) complexes with Shep I were calculated on the basis of the titration curves recorded in the pH range of 2.50–10.00 (Figure S6). The pH-dependent complex species are described in detail in the SI and summarized in Table S3.

**Figure 3:**
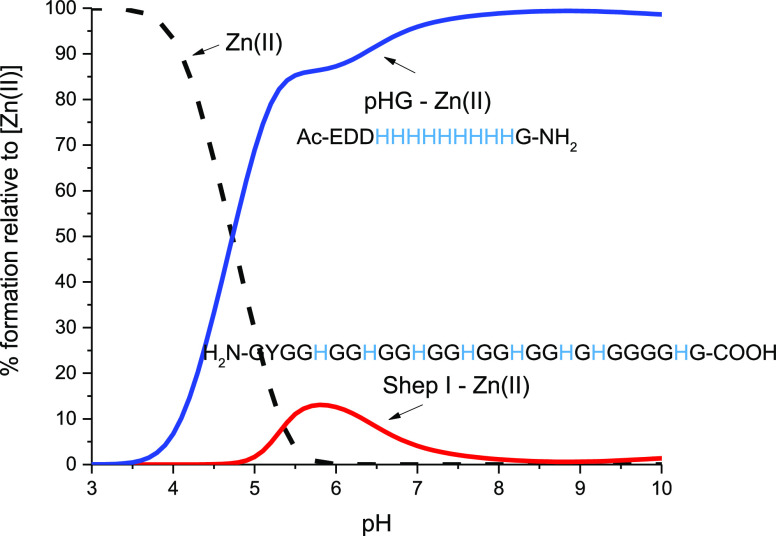
Competition plots for the Zn(II) complexes with Shep I (red) and pHG (blue; Ac-EDDHHHHHHHHHG-NH_2_) peptides based on potentiometric data (Table 1 and for pHG taken from ref (45)).

At physiological pH, where the [ZnH_3_L]^4*+*^ species dominate in solution, zinc(II) is bound by up to four histidine imidazoles (suggested on the basis of potentiometric titrations, Figure S6 and Table S3). It is also possible that the so-called polymorphic forms may occur in these complex species, where zinc(II) is bound to two different sets of {2N_im_} that are in equilibrium (similar to the Shep I–Cu(II) complex species at pH around 7.40). However, such a coordination mode is also definitely less stable (as in the case of Cu(II) complexes) than that of the complex with the pHG peptide (Figure 3), in which His residues are not separated and bind zinc ion by different donor sets of {3N_im_}.^44,45^

### Impact of Metal Coordination on Shepherin I Structure

**Figure 4:**
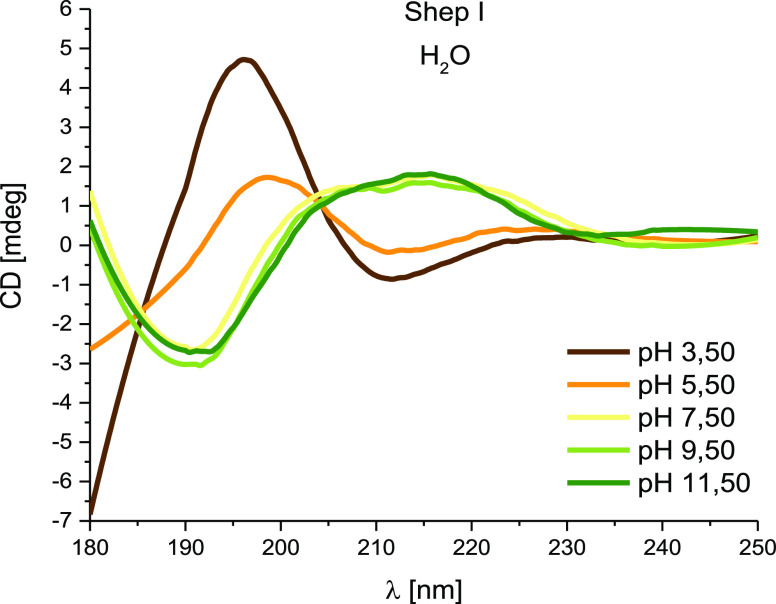
Far-UV CD spectra for Shep I in aqueous solution of 4 mM HClO_4_ with *I* = 0.1 M NaClO_4_ strongly depend on pH; optical path length = 0.1 mm. *C*_L_ = 0.3 mM.

Far-UV CD spectra of shepherin I show a β-sheet conformation at acidic pH (pH 3.50, and 5.50) (Figure 4). Above pH 7.50, characteristic spectra for random coils were observed.

**Figure 5:**
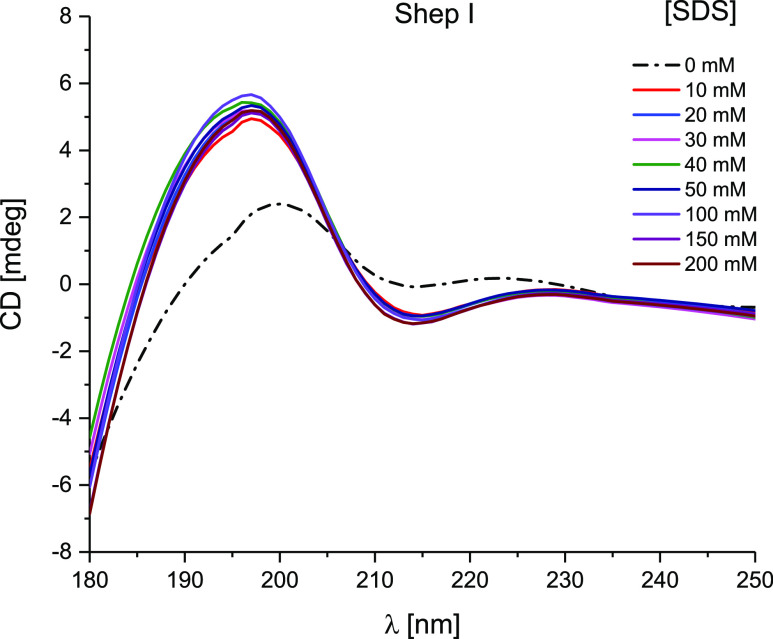
Far-UV CD spectra for Shep I in aqueous solution of 4 mM HClO_4_ titrated by addition of SDS solution at pH 5.50; optical path length = 0.2 mm. *C*_L_ = 0.3 mM.

An interesting phenomenon was observed when Shep I was titrated with SDS at pH 5.50–quite surprisingly, the presence of 10 mM SDS increases the amount of β-sheet conformation, instead of inducing a structural rearrangement to an α-helical structure, what would have been expected from the helix-inducing SDS.^49^ The addition of additional SDS equivalents did not affect the structure (Figure 5).

**Figure 6:**
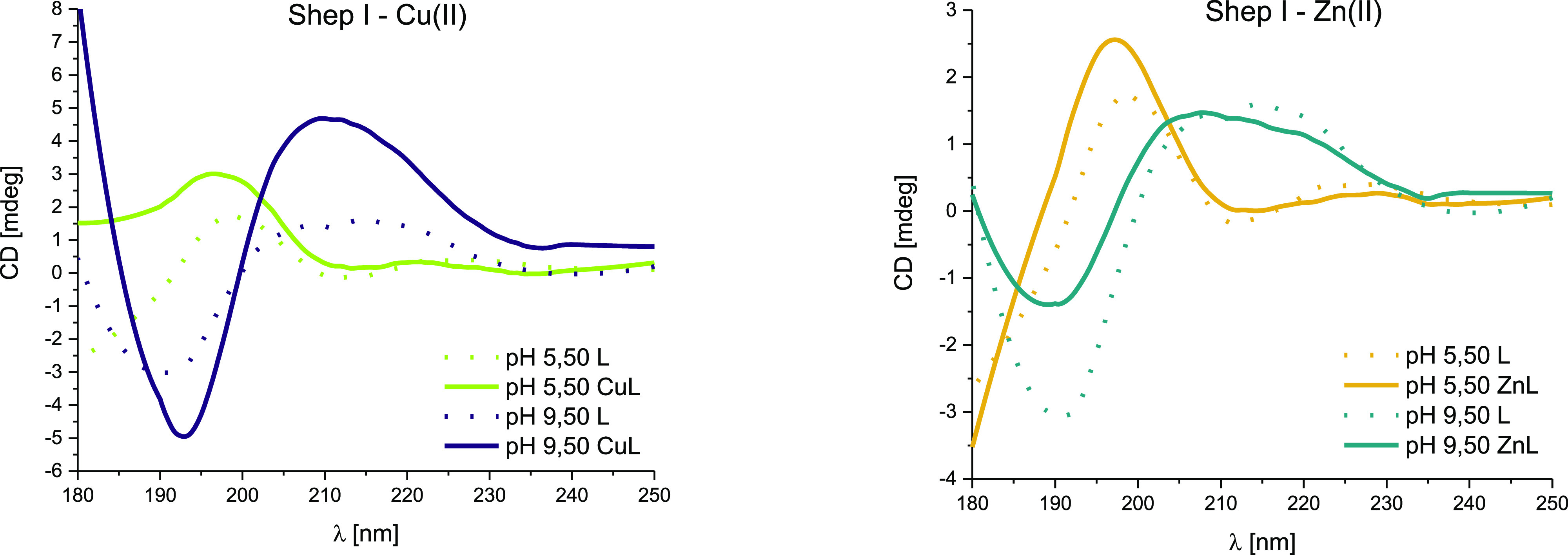
Comparison of far-UV CD spectra at 180–250 nm for shepherin I–metal complexes at acidic (5.50) and alkaline (9.50) pH in aqueous solution of 4 mM HClO_4_ with *I* = 0.1 M NaClO_4_; molar ratio M/L 0.9:1; the optical path length = 0.1 mm; C_L_ = 0.3 mM. The dotted lines correspond to the spectra for the peptide.

Both Cu(II) and Zn(II) slightly enhance the amount of the β-sheet structure in Shep I at acidic pH (Figure 6). At alkaline pH, random coil conformations were observed in both complexes. These results prove that the pH value, and not the addition of metal ions, is crucial for Shep I secondary structure.

In the presence of metal ions at pH 5.50, the positive maximum at ca. 197 nm became much more intense and may suggest the β-pleated sheet(s) stabilization (Figure 6, Table S4). However, the differences in the wavelength for the second maximum (shift from 222.8 to 228.8 nm, Table S4) may suggest that there may be (slightly) different conformations.^23^

At physiological pH, a mild turbidity was observed in potentiometric and spectroscopic studies; that is why, in order to check the structure/morphology of the metal complexes at pH 7.40 AFM imaging was used (discussed later in the text).

### Antimicrobial Activity

Do the thermodynamic stabilities and structural changes correlate with the antimicrobial properties of Shep I and its metal complexes? To answer this question, we carried out broth microdilution and TTC reduction tests. Both methods allowed determination of MIC_50_, representing the minimum inhibitory concentration at which microbial growth is inhibited for 50% of the tested microorganisms, as well as the MBC, which corresponds to the lowest concentration of a compound/complex required to eradicate a specific microorganism. The study encompassed six bacterial strains and one fungal strain. The comprehensive results of the antimicrobial activity assays are presented in Table 2, showing that, very surprisingly, the only compound for which any biological activity was detected was the Shep I–Zn(II) complex.

**Table 2:** *In Vitro* Antimicrobial Activity of Shepherin I with or without Cu(II)/Zn(II) Ions, Presented as MIC_50_ [μg/mL]^a^

	*C. albicans* ATCC 10231	*E. coli* ATCC 25922	*MRSA* ATCC 43300	*P. aeruginosa* ATCC 27853	*E. faecalis* ATCC 29212	*K. pneumoniae* ATCC 700603	*A. baumannii* ATCC 19606
Shep I	n/d	n/d	n/d	n/d	n/d	n/d	n/d
Shep I–Cu(II)	n/d	n/d	n/d	n/d	n/d	n/d	n/d
Shep I–Zn(II)	**32**	n/d	n/d	n/d	n/d	n/d	n/d

a Significant values are bolded (n/d–not detected).

Shepherin I, in combination with zinc(II) ions, exhibits robust antifungal activity. The MIC_50_ values for Shep I–Zn(II) were determined to be 32 μg/mL. No antibacterial activity was observed within the tested concentration range, neither for shepherin I, nor for its complexes with metal ions.

Based on the antimicrobial activity results, the cytotoxicity of shepherin I in combination with zinc(II) ions was evaluated against human primary renal proximal tubule epithelial cells (RPTEC). The neutral red (NR) uptake assay was employed, which relies on the ability of living cells to incorporate and bind NR in their lysosomes. The cytotoxicity of the peptide-metal complex was evaluated at two time points: after 48 and 72 h of incubation with the cells. The results obtained from these experiments are presented in Table 3.

**Table 3:** Cytotoxicity [%] of Shep I–Zn(II) after 48/72 h of Incubation with RPTEC Cell Line^a^

		cytotoxicity [%]	
	conc. [μM]	48 h	72 h
Shep I–Zn(II)	125	12	18
	75	9	7
	10	9	0
	1	7	0

a Determined using the neutral red (NR) uptake assay.

The cytotoxicity assessment reveals that Shep I in combination with zinc(II) ions does not exhibit any significant cytotoxic effects on the RPTEC cell line within the tested concentration range. This finding suggests that the investigated peptide-metal ion complex, which has demonstrated potent antifungal activities, holds promise as a potential therapeutic agent with a favorable safety profile, exhibiting low cytotoxicity toward normal cellular function. The observed cytotoxicity level does not hinder the practical application of this peptide-metal complex.

These findings highlight the potential of Shep I in combination with zinc(II) ions as a promising effective agent for antifungal interventions. We were intrigued to find out why this particular complex, and not the copper(II) complex or the free ligand itself, shows very reasonable antifungal activity. We decided to take a literal “closer look” at this phenomenon, highlighting morphological changes that occur after Zn(II) is bound to Shep I under an atomic force microscope.

### Morphology

**Figure 7:**
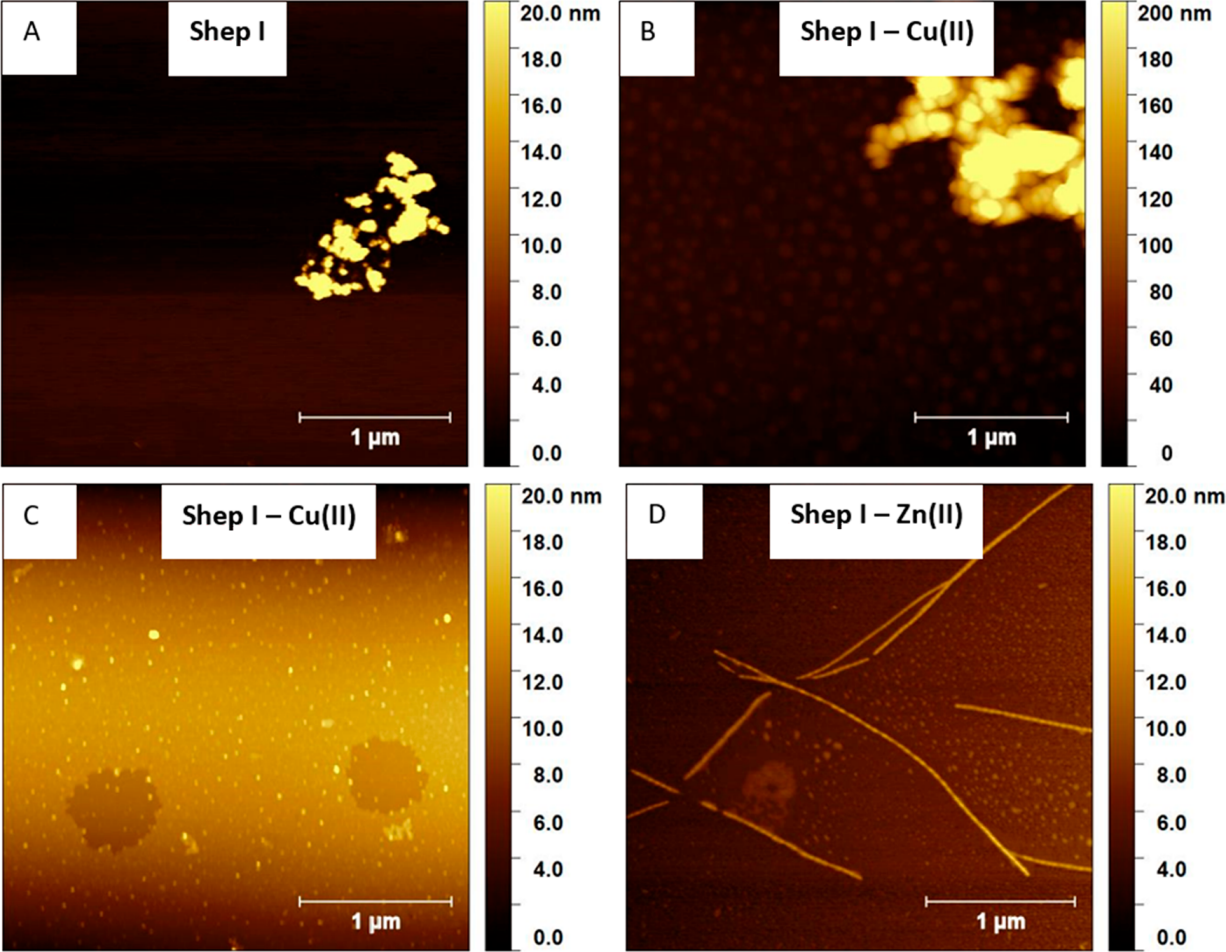
AFM images of Shep I and its complexes with Cu(II) and Zn(II) ions at pH 7.4.

We analyzed the morphology of Shep I and its complexes with Cu(II) and Zn(II). The complexes became mildly turbid at pH 7.4, contrary to the free Shep I sample. Directly after preparation of the Shep I sample, AFM images showed the presence of aggregates that preferred to be associated together instead of being separated (Figure 7A) Similar aggregates were found in Shep I complexes with Cu(II), both in associated and separated forms (Figure 7B,C). Interestingly, for Shep I complexes with Zn(II), already at the initial time point, amyloid fibrils were visible with a mean width of 28.3 ± 5.1 nm and mean height of 6.1 ± 0.9 nm, with crossover distance equal to 46.0 ± 5.0 nm (Figure 7D).

These observations show that coordination of Zn(II) to Shep I triggers the formation of fibrillar structures, in contrast to Shep I alone or to its complex with Cu(II). This finding is in perfect agreement with the antifungal properties of the Shep I–Zn(II) complex, suggesting a correlation between the complex morphology and antimicrobial activity.

## Conclusion

It is estimated that around 2 million people worldwide die each year as a result of fungal infections. *C. albicans* is the main pathogenic opportunistic fungus in humans and the most serious complication caused by this pathogen is systemic candidiasis, characterized by high mortality and neutropenia.^50^ In recent years, candidiasis in hospitalized patients infected with the SARS-CoV-2 virus (hospital secondary infection) has also become a severe problem.^51^

Commonly used antifungal drugs such as triazoles, pyrimidine analogues, echinocandins, and polyenes become less and less effective, especially when the infection involves *C. albicans* biofilm,^52^ therefore new drugs are being intensively sought. Commercially available groups of antifungal peptides include 1,3-β-glucan synthesis inhibitors (among them are the commercially available caspofungin, anidulafungin, and micafungin), cell wall chitin inhibitors, peptides which disrupt the fungal membrane and peptides that use more than one mode of action.^53^ The Shep I–Zn(II) complex, with a fibril-linked mode of action, may become a promising agent in the fight against candidiasis, provided that further studies elucidate the precise underlying mechanisms of their most likely fibril-related antifungal action.

Once again, it becomes clear that there is a significant and underestimated effect of metal coordination on antimicrobial activity. Often, metal binding causes a morphological and/or structural change that triggers a different mode of action, enhancing, or even initiating antimicrobial properties of AMPs.

Recently, we have described a similar effect of Zn(II)-triggered structural change of the antidiabetic (and also antifungal) pramlintide.^16^ In that case, binding of Zn(II) to the N-terminal amine group and to the imidazole of His18 resulted in a kink of the peptide, which triggered the formation of fibrils, which turned out to have antifungal properties. Although the phenomenon itself was fascinating, the applicable potential of such a complex (with a 256 μM MIC_50_) was marginal. In the case of shepherin I, we observe a similar scheme of Zn(II) coordination–structural change–fibril formation–anticandidal activity; however, in this case, a considerable potential is definitely present (32 μM MIC_50_). To the best of our knowledge, in the literature, there is very little information both on (i) how the nature and location of Zn(II) binding residues influence the properties of fibril self-assembly^54^ as well as on (ii) the antifungal mode of action of amyloid fibrils; they may, as in the case of serum albumin amyloid, disrupt the fungal membrane or cell wall, via interactions with the candidal Als3 cell wall protein.^55^ Further investigations that aim to explain the mechanism of the fibrils being antifungal and to explore their potential against other clinically relevant fungal strains are absolutely necessary.

## Supporting Information Available

pH-dependent species distributions, distribution diagrams for Shep I–Cu(II) and Shep I–Zn(II) systems in aqueous solution of 4 mM HClO_4_, pH-dependent spectra, *m*/*z* values for Shep I ligand and complexes with Cu(II) and Zn(II) ions, mass spectra (ESI-MS), pH-dependent spectra, potentiometric and spectroscopic data for proton and Shep I–Cu(II) system, Stability constants, positive band maxima in far-UV CD spectroscopy and a detailed description of the coordination of Cu(II) and Zn(II) ions with Shep I (PDF)
