# Supplementary material for: Zn(II) Induces Fibril Formation and Antifungal Activity in Shepherin I, An Antimicrobial Peptide from Capsella bursa-pastoris
Source: Inorg Chem. 2023 Nov 20;62(48):19786–94. doi: 10.1021/acs.inorgchem.3c03409 (PMC10698721; doi:10.1021/acs.inorgchem.3c03409)
Supplement: Supplementary file 1 — ic3c03409_si_001.pdf [file ic3c03409_si_001.pdf]

# Supporting information for:

# Zn(II) induces fibril formation and antifungal activity in shepherin I, an antimicrobial peptide from *Capsella bursa pastoris*

*Joanna Wątły,*,† Klaudia Szarszoń,† Aleksandra Mikołajczyk,‡ Manuela Grelich-Mucha,§ Agnieszka Matera-Witkiewicz,‡ Joanna Olesiak-Bańska,§ and Magdalena Rowińska-Żyrek*,†*

†Faculty of Chemistry, University of Wrocław, F. Joliot-Curie 14, 50-383 Wrocław, Poland ‡Screening of Biological Activity Assays and Collection of Biological Material Laboratory, Wrocław Medical University Biobank, Faculty of Pharmacy, Wrocław Medical University, 50-556 Wrocław, Poland §Faculty of Chemistry, Wrocław University of Science and Technology, Wyb. Wyspiańskiego 27, 50-370, Wrocław, Poland

TABLE OF CONTENTS

Figure S 1. pH-dependent Shep I species distribution in aqueous solution of 4 mM $HClO_4$ with $I$ = 0,1 M $NaClO_4$ at 298 K. $C_L$ = 0,5 mM. ........ 3

Table S 1. *m/z* values for Shep I ligand and complexes with Cu(II) and Zn(II) ions observed in experimental mass spectrometry. [L]=0,0001 M; M:L molar ratio 1:1; pH 6; solvent methanol:water 50:50. ........ 3

Figure S 2. Mass spectra (ESI-MS) for the system Shep I – Cu(II) A) whole spectrum with ; B) example of experimental and simulated spectrum for $[CuL]^{3+}$. Additional signals are mainly chloride and sodium adducts of ligand and complex species, as well as minor instrument impurities. ........................................4

Figure S 3. Mass spectra (ESI-MS, Bruker Apex FT-ICR spectrometer) for the system Shep I – Zn(II) A) whole spectrum with example of experimental and simulated spectrum for $[L]^{4+}$ ligand; B) – D) examples of experimental and simulated spectrum for $[ZnL]^{3+}$, $[ZnL]^{4+}$, $[ZnL]^{5+}$, respectively. Additional signals are mainly chloride and sodium adducts of ligand and complex species, as well as minor instrument impurities ..........................................................................................................................5

Figure S 4. Distribution diagram for the Shep I - Cu(II) system in aqueous solution of 4 mM $HClO_4$ with $I$ = 0,1 M $NaClO_4$ dependent on pH values at 298 K. $C_L$ = 0,5 mM; molar ratio [L] : [Cu(II)] = 1 : 0,9. ....6

Figure S 5. pH- dependent spectra: A) UV-vis and B) CD for the Shep I – Cu(II) system in aqueous solution of 4 mM $HClO_4$ with $I$ = 0,1 M $NaClO_4$. Optical path lengths of 1 cm. $C_L$ = 0,4 mM; [L] : [Cu(II)] = 1:0,9. ...........................................................................................................................7

Table S 2. Potentiometric and spectroscopic data for proton and Shep I – Cu(II) system in aqueous solution of 4 mM $HClO_4$ for each calculated complex species with the proposed coordination modes. $C_L$ = 0,3 - 0,5 mM; molar ratio [L] : [Cu(II)] = 1 : 0,9 ; $I$ = 0,1 M $NaClO_4$; T = 298 K. ......................................8

Figure S 6. Distribution diagram for the Shep I - Zn(II) system in aqueous solution of 4 mM $HClO_4$ with $I$ = 0,1 M $NaClO_4$ dependent on pH values at 298 K. $C_L$ = 0,5 mM; molar ratio [L] : [Zn(II)] = 1 : 0,9. ....10

Table S 3. Stability constants for Shep I - Zn(II) system in aqueous solution of 4 mM $HClO_4$ for each calculated complex species with the proposed coordination modes. $C_L$ = 0,5 mM; [L] : [Zn(II)] = 1 : 0,9 ; $I$ = 0,1 M $NaClO_4$; T = 298 K. ..........................................................................................................11

Table S 4. Positive band maxima in far-UV CD spectroscopy in water solution of 4 mM $HClO_4$ with $I$ = 0,1 M at pH 5,50. ..........................................................................................................................11

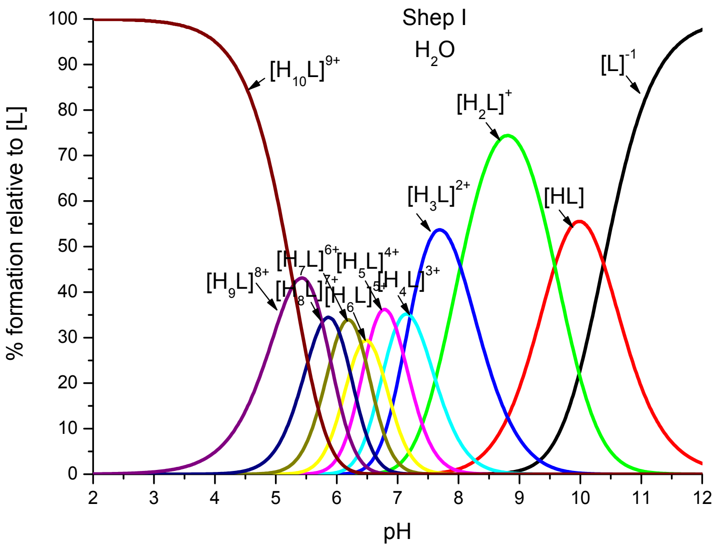


Figure S 1. pH-dependent Shep I species distribution in aqueous solution of 4 mM $HClO_4$ with $I$ = 0,1 M $NaClO_4$ at 298 K. $C_L$ = 0,5 mM.

Table S 1. *m/z* values for Shep I ligand and complexes with Cu(II) and Zn(II) ions observed in experimental mass spectrometry. [L] = 0,0001 M; M:L molar ratio 1:1; pH 6; solvent methanol:water 50:50.

| **Shep I – Cu(II)** | | **Shep I – Zn(II)** | |
|---|---|---|---|
| Species | Signal at *(m/z)* | Species | Signal at *(m/z)* |
| $[L]^{2+}$ | 1181,98 | | |
| $[L]^{3+}$ | 788,32 | $[L]^{3+}$ | 787,95 |
| $[L]^{4+}$ | 591,50 | $[L]^{4+}$ | 591,22 |
| $[L]^{5+}$ | 473,30 | $[L]^{5+}$ | 473,18 |
| $[L]^{6+}$ | 394,67 | | |
| $[L]^{7+}$ | 338,43 | | |
| $[CuL]^{3+}$ | 808,30 | $[ZnL]^{3+}$ | 809,61 |
| $[CuL]^{4+}$ | 605,50 | $[ZnL]^{4+}$ | 606,94 |
| $[CuL]^{5+}$ | 484,80 | $[ZnL]^{5+}$ | 485,58 |
| $[CuL]^{6+}$ | 405,00 | | |

Shep I – Cu(II)

A

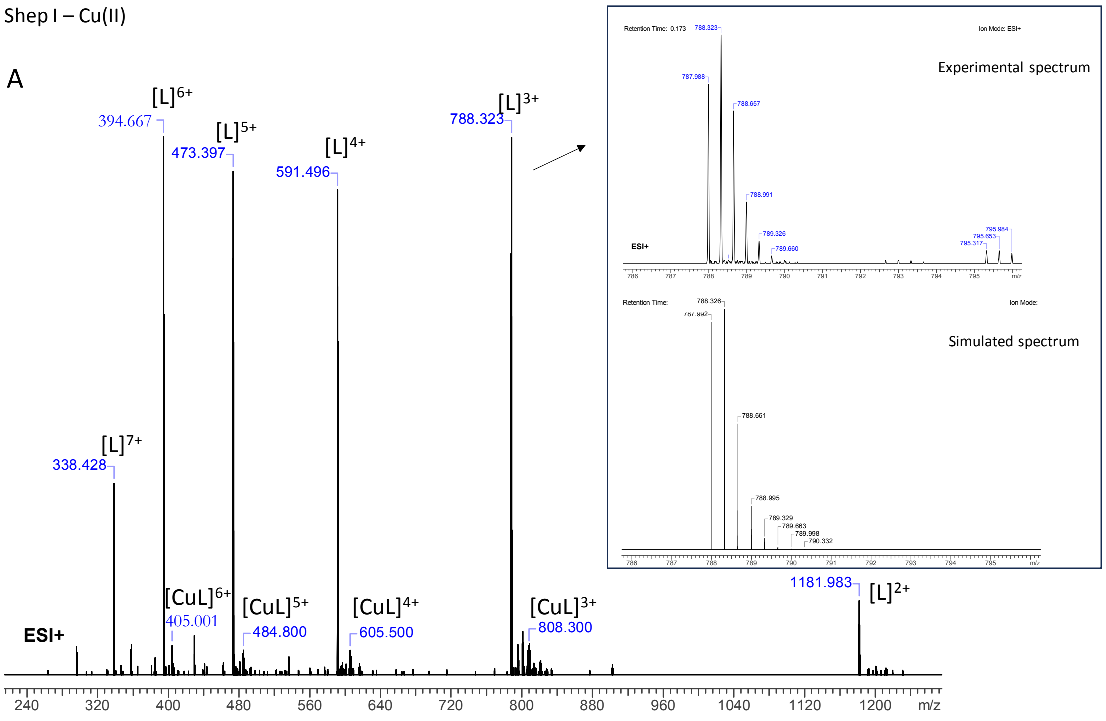


B [CuL]$^{3+}$

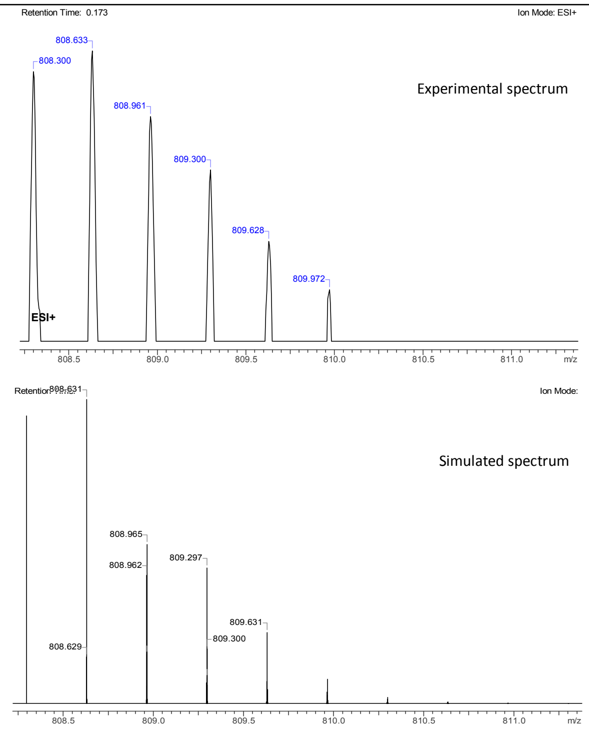

Figure S 2. Mass spectra (ESI-MS, Shimadzu q-TOF LCMS 9030) for the system Shep I – Cu(II) A) whole spectrum with example of experimental and simulated spectrum for $[L]^{3+}$ ligand; B) example of experimental and simulated spectrum for $[CuL]^{3+}$. Additional signals are mainly chloride and sodium adducts of ligand and complex species, as well as minor instrument impurities.

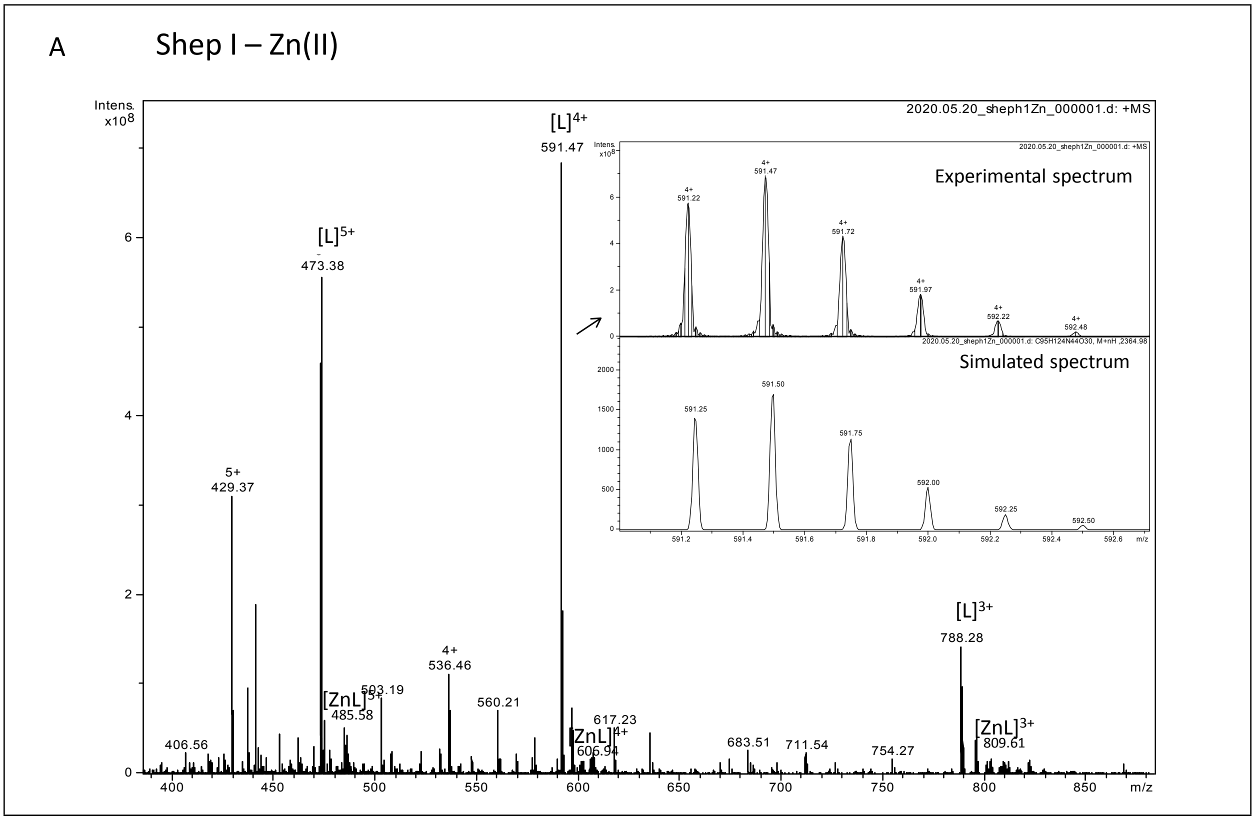

B [ZnL]$^{3+}$

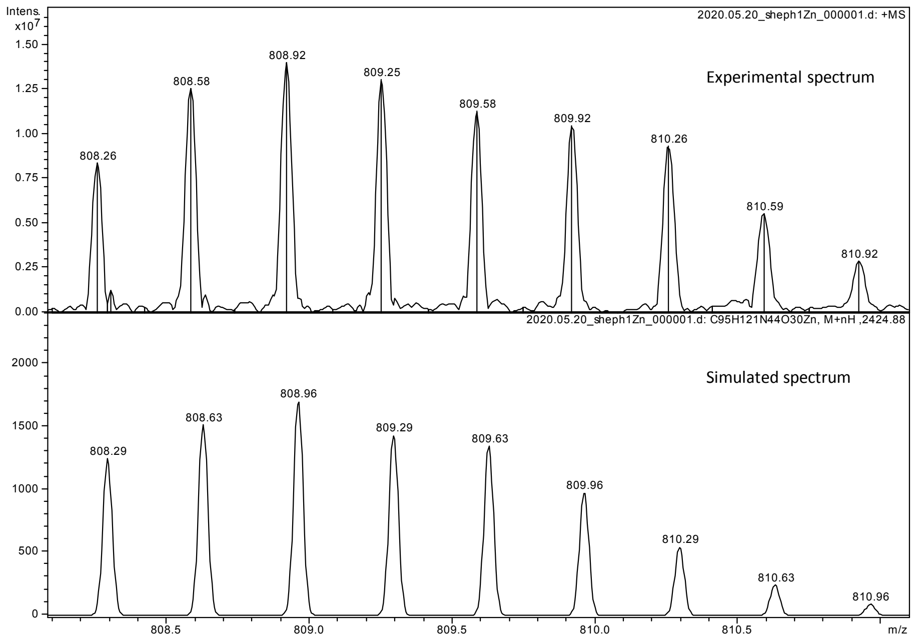
Intens.
x10^7
2020.05.20_sheph1Zn_000001.d: +MS
Experimental spectrum
808.26
808.58
808.92
809.25
809.58
809.92
810.26
810.59
810.92
2020.05.20_sheph1Zn_000001.d: C95H121N44O30Zn, M+nH ,2424.88
Simulated spectrum
808.29
808.63
808.96
809.29
809.63
809.96
810.29
810.63
810.96
m/z

C [ZnL]$^{4+}$

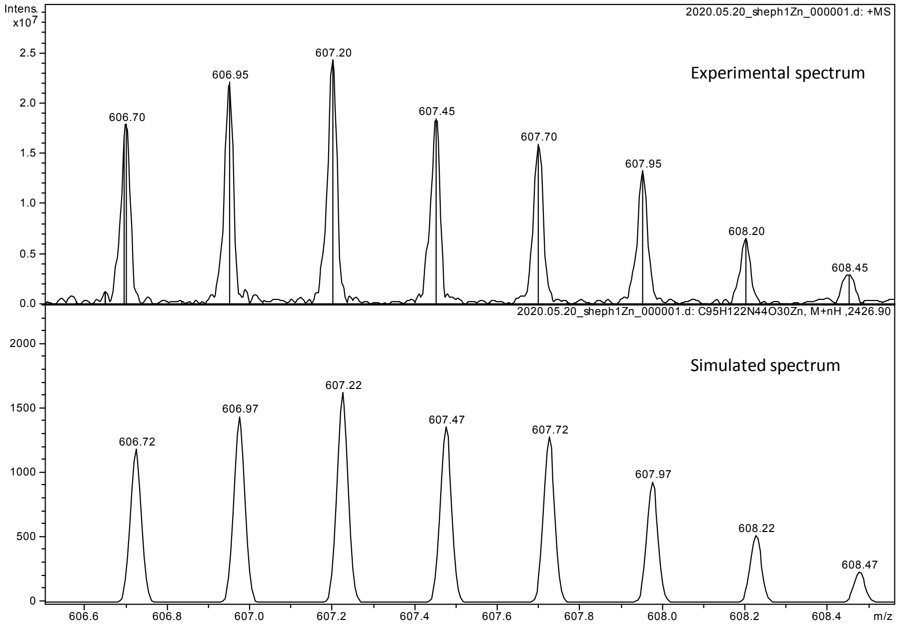
Intens.
x10^7
2020.05.20_sheph1Zn_000001.d: +MS
Experimental spectrum
606.70
606.95
607.20
607.45
607.70
607.95
608.20
608.45
2020.05.20_sheph1Zn_000001.d: C95H122N44O30Zn, M+nH ,2426.90
Simulated spectrum
606.72
606.97
607.22
607.47
607.72
607.97
608.22
608.47
m/z

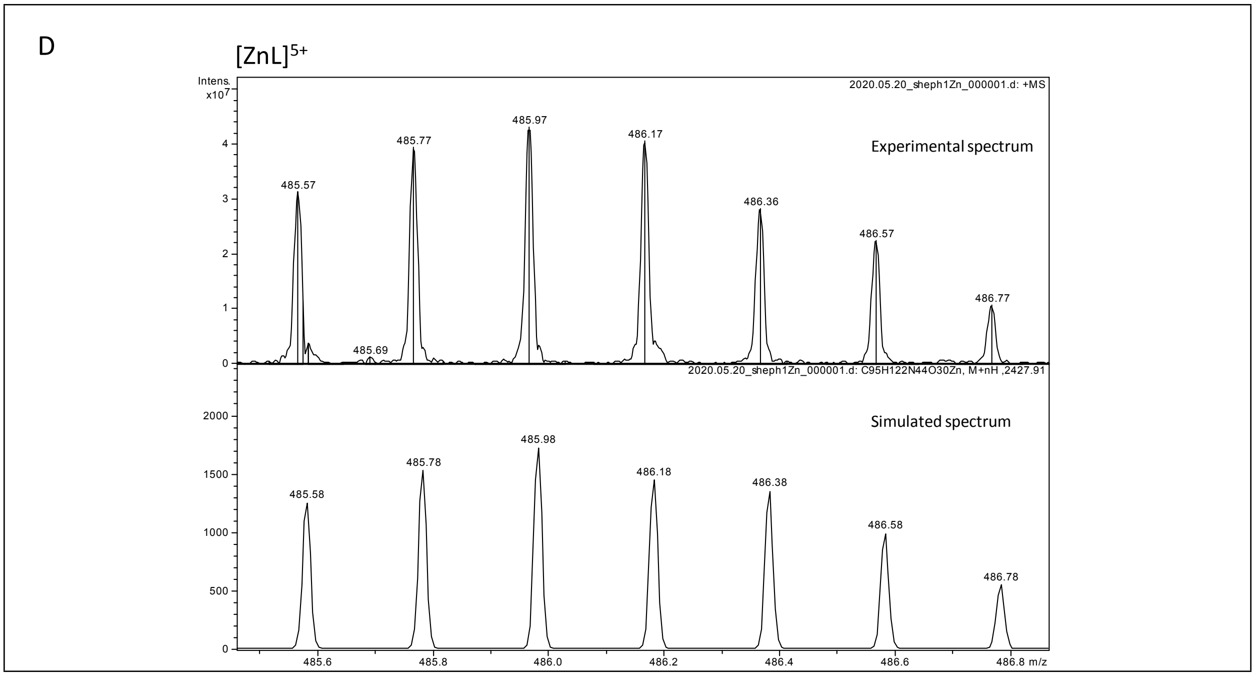


Figure S 3. Mass spectra (ESI-MS, Bruker Apex FT-ICR spectrometer) for the system Shep I – Zn(II) A) whole spectrum with example of experimental and simulated spectrum for $[L]^{4+}$ ligand; B) – D) examples of experimental and simulated spectrum for $[ZnL]^{3+}$, $[ZnL]^{4+}$, $[ZnL]^{5+}$, respectively. Additional signals are mainly chloride and sodium adducts of ligand and complex species, as well as minor instrument impurities.

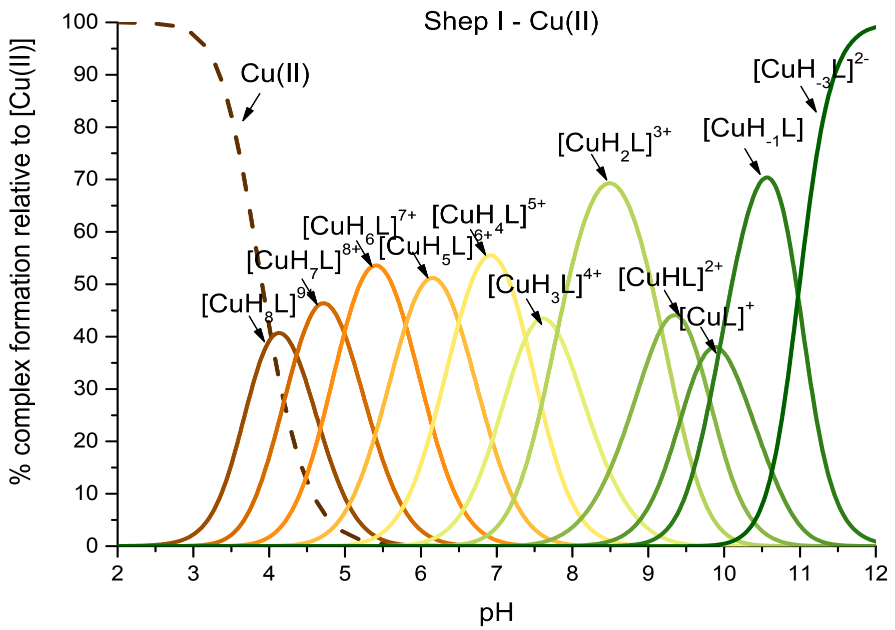


Figure S 4. Distribution diagram for the Shep I - Cu(II) system in aqueous solution of 4 mM $HClO_4$ with $I$ = 0,1 M $NaClO_4$ dependent on pH values at 298 K. $C_L$ = 0,5 mM; molar ratio [L] : [Cu(II)] = 1 : 0,9.

The Shep I peptide starts to bind Cu(II) ions at pH around 3Figure S 4. Distribution diagram for the Shep I - Cu(II) system in aqueous solution of 4 mM $HClO_4$ with $I$ = 0,1 M $NaClO_4$ dependent on pH values at 298 K. $C_L$ = 0,5 mM; molar ratio [L] : [Cu(II)] = 1 : 0,9.

. The first complex species is $[CuH_8L]^{9+}$, with a maximum concentration at pH 4,10. The presence of d-d transition band with a maximum absorption at 710 nm a in the UV-vis spectra and as well as a band with a positive Cotton effect in the circular dichroism spectra at $\lambda_{max}$ 250 nm (Figure S5) suggest the coordination of one imidazole nitrogen at this pH.

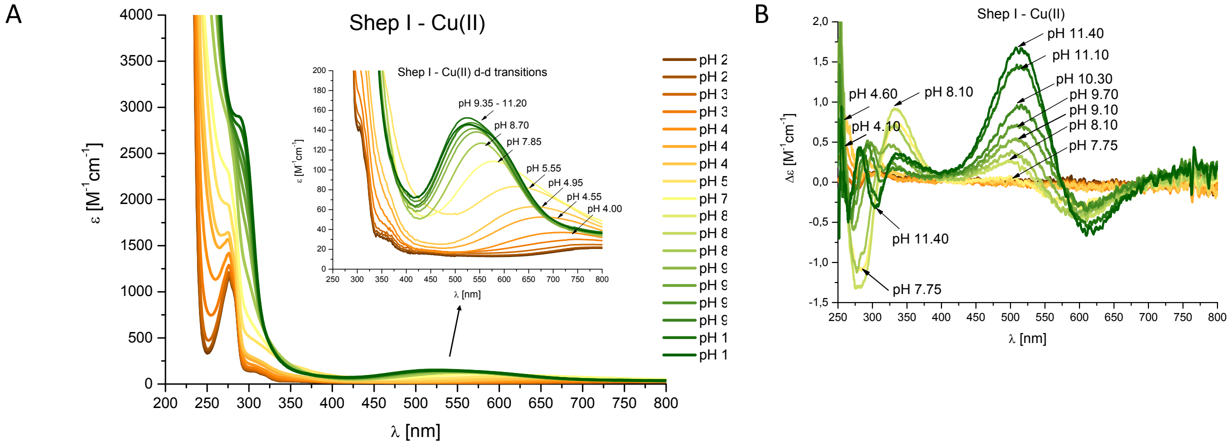


Figure S 5. pH- dependent spectra: A) UV-vis and B) CD for the Shep I – Cu(II) system in aqueous solution of 4 mM $HClO_4$ with $I$ = 0,1 M $NaClO_4$. Optical path lengths of 1 cm. $C_L$ = 0,4 mM; [L] : [Cu(II)] = 1 : 0,9.

The spectroscopic results for the next complex species, $[CuH_7L]^{8+}$ ($\lambda_{max}$ = 678 nm in the UV-vis spectra and a band in the CD spectrum similar to that observed with the previous complex (Figure S5) are typical for a {$2N_{im}$} donor set, which is in good agreement with a significant decrease in p$K_a$ value from 6,04 (in the free peptide) to 4,36 (in the complex) which suggests the coordination of the next imidazole nitrogen. A situation in which at least two complex species exist in equilibrium, in which 2 different nitrogen atoms are coordinated to Cu(II). Most likely, these phenomena also take place in the $[CuH_6L]^{7+}$ complex species: in the UV-vis spectra, slight shifts of the absorption maximum towards shorter wavelengths were observed, from 678 nm to 630 nm at pH 5,55, but still indicating only a {2N} donor set, what was also observed in our previously studied His-rich – Cu(II) systems and has been called polymorphic binding sites (metal can ‘‘move back and forth” along such regions). In case of Shep I peptide, the regularly repeating GGH motif (GGHGGHGGHGGHGGHGGH) is an excellent motif for this type of metal ion binding (histidines separated by two amino acids).[1,2] Mild turbidity of the complex solution was observed at pH about 5,60 - 7,70, most likely due to the accumulation of a positive charge caused by the deprotonation of the next histidyl residues. The precise analysis of the coordination sphere in the next four complex species, $[CuH_5L]^{6+}$, $[CuH_4L]^{5+}$, $[CuH_3L]^{4+}$ and also $[CuH_2L]^{3+}$ is much more difficult due to lack of reliable

spectroscopic data. However, the p$K_a$ values strongly suggest non-bonded deprotonation of the next imidazole residues (with p$K_a$ = 6,54, p$K_a$ = 6,97, p$K_a$ =7,21 and p$K_a$ = 8,03, respectively) and still the occurrence of polymorphic binding states with {$2N_{im}$} donor set in the studied system.

Above pH 7,70, the solution becomes clear and a new bands in the CD spectrum appear with a minor positive (at 500 nm) and clear negative (at 603 nm) Cotton effects and charge transfer band $N^-$ → Cu(II) at 330 nm, which suggest coordination of an amide nitrogen (Figure S5 B). UV-vis spectrum also supports the coordination of the next nitrogen atom by blue shift of maximum band (from 630 nm to 576 nm in the range of pH 5,55 – 7,85) and suggests a {$2N_{im}$,$1N_{am}$} donor set for $[CuHL]^{2+}$ (Figure S5 A).

The next complex species, $[CuL]^+$, is formed as a result of deprotonation of the non-bonding N-terminal amine, as evidenced by the similar values of p$K_a$ = 9,58 for the ($[H_2L]^+$ species) and p$K_a$ = 9,68 for the complex ($[CuL]^+$ species) and no significant changes in the spectral parameters suggest the same donor set {$2N_{im}$, $1N_{am}$}.

Above pH 9, when $[CuH_{-1}L]$ complex species begin to form, further changes in the spectroscopic parameters were observed: (i) shift of the maximum absorption to shorter wavelengths (556 nm → 536 nm at pH 9,35) in the UV-vis spectrum and (ii) slight shift of Cotton effect maxima and significant increase of their intensity in CD spectrum (Figure S5), suggesting the coordination of the next amide nitrogen resulted in {4N} coordination.

With an increase in pH (above pH 11), small hypsochromic shifts (from 536 nm to 524 nm) with a hyperchromic effect are observed in the UV-vis spectrum, and the studied solution of the complex turns into a slightly violet colour, which indicates the presence of a copper(II) complex with a typical 4N square planar geometry with a {$1N_{im}$, $3N_{am}$} donor set for the last complex species, $[CuH_{-3}L]^{2-}$ (the CD spectrum with clear signals with a positive value of the Cotton effect at $\lambda_{max}$ = 515 nm ($\Delta\varepsilon$ = 1,45) and a negative value at $\lambda_{max}$ = 613 nm ($\Delta\varepsilon$ = -0,55)). This complex is formed by the replacement of one of the one imidazole nitrogen atom with a third amide nitrogen in the coordination sphere of copper(II) ions.

Table S 2. Potentiometric and spectroscopic data for proton and Shep I – Cu(II) system in aqueous solution of 4 mM $HClO_4$ for each calculated complex species with the proposed coordination modes. $C_L$ = 0,3 - 0,5 mM; molar ratio [L] : [Cu(II)] = 1 : 0,9 ; $I$ = 0,1 M $NaClO_4$; T = 298 K.

| | Potentiometry | | | UV-vis | | | CD | | | Proposed coordination modes |
|---|---|---|---|---|---|---|---|---|---|---|
| Complex species | log $\beta$ | p$K_a$ | pH | nm | ε [$M^{-1}$ $cm^{-1}$] | | nm | Δε [$M^{-1}$ $cm^{-1}$] | | |
| **Shep I – Cu(II)** | | | | | | | | | | |
| **$[CuH_8L]^{9+}$** | 67,57(5) | | 4,10 | 276<br>710 | 1419<br>37,03 | | 251 | 0,69 | | 1N {1x$N_{im}$} |
| **$[CuH_7L]^{8+}$** | 63,21(2) | 4,36 | 4,70 | 276<br>678 | 15868<br>52,52 | | 251 | 0,83 | | 2N {2x$N_{im}$}<br>(potentiometry: 3$N_{im}$)<br>'polymorphic binding sites' |
| **$[CuH_6L]^{7+}$** | 58,22(2) | 4,99 | 5,40 | 276<br>630 | 1934<br>83,19 | | 251 | 0,88 | | 2N {2x$N_{im}$}<br>(potentiometry: 4$N_{im}$)<br>'polymorphic binding sites' |
| **$[CuH_5L]^{6+}$** | 52,42(2) | 5,80 | 6,15 | mild turbidity | | | mild turbidity | | | 2N {2x$N_{im}$}<br>Deprotonation of non-bonding His residues<br>(with p$K_a$ 6,54, p$K_a$ 6,97, p$K_a$ 7,21 and p$K_a$ 8,03) |
| **$[CuH_4L]^{5+}$** | 45,92(2) | 6,50 | 6,90 | | | | | | | |
| **$[CuH_3L]^{4+}$** | 38,54(3) | 7,38 | 7,60 | | | | | | | |
| **$[CuH_2L]^{3+}$** | 30,74(3) | 7,80 | 8,50 | | | | | | | |
| **$[CuHL]^{2+}$** | 21,57(4) | 9,17 | 9,35 | 576 | 108,67 | | 277<br>333<br>500<br>601 | -1,13<br>0,72<br>0,06<br>-0,33 | | 3N {2x$N_{im}$, 1x$N_{am}$} |
| **$[CuL]^{+}$** | 11,89(4) | 9,68 | 9,90 | 556 | 126,96 | | 277<br>333<br>501<br>602 | -1,31<br>0,91<br>0,27<br>-0,48 | | 3N {2x$N_{im}$, 1x$N_{am}$}<br>Deprotonation of non-bonding N-terminal $NH_2$<br>(with p$K_a$ 9,58) |
| **$[CuH_{-1}L]$** | 1,95(3) | 9,94 | 10,60 | 536 | 141,57 | | 270<br>298<br>316<br>511<br>612 | -0,51<br>0,50<br>0,14<br>0,70<br>-0,37 | | 4N {2x$N_{im}$, 2x$N_{am}$} |

| | | | | | | | | | | |
|---|---|---|---|---|---|---|---|---|---|---|
| **$[CuH_{-3}L]^{2-}$** | -20,00(3) | | 11,90 | 289<br>524 | 2886<br>145,98 | | 265<br>281<br>303<br>333<br>515<br>613 | -050<br>0,43<br>-0,32<br>0,36<br>1,45<br>-0,55 | | 4N {1xN$_{im}$, 3xN$_{am}$}<br>Deprotonation of Tyr residue<br>(with p$K_a$ 10,38) |

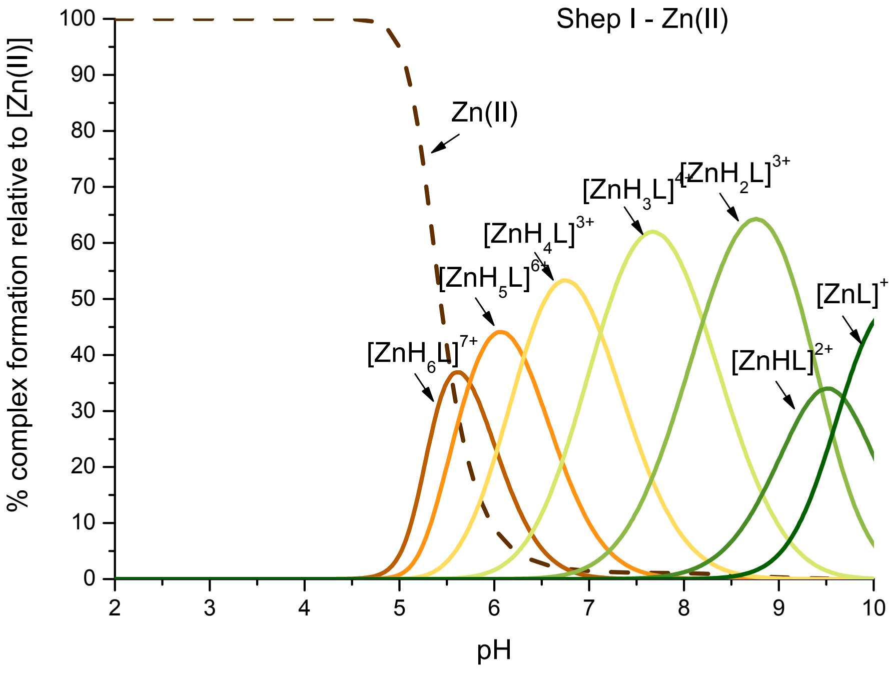


Figure S 6. Distribution diagram for the Shep I - Zn(II) system in aqueous solution of 4 mM $HClO_4$ with $I$ = 0,1 M $NaClO_4$ dependent on pH values at 298 K. $C_L$ = 0,5 mM; molar ratio [L] : [Zn(II)] = 1 : 0,9.

In the first complex detected at acidic pH, $[ZnH_6L]^{7+}$, with a maximum concentration at pH 5,50, Zn(II) is probably already bound to up to four nitrogen atoms from four histidyl residues, resulting in {4N$_{im}$} binding mode. It is also quite possible that polymorphic forms may occur for this complex species, where zinc ion is bound by two different sets of {2N$_{im}$}. The suggested coordination mode is the same in case for the following species: $[ZnH_5L]^{6+}$, $[ZnH_4L]^{5+}$, $[ZnH_3L]^{4+}$, $[ZnH_2L]^{3+}$, $[ZnHL]^{2+}$ and $[ZnL]^{+}$. The p$K_a$ values assigned to the mentioned complexes are derived from non-bonding deprotonations of: (i) imidazole group

from four His residues; (ii) $NH_2$ from the peptide terminus and (iii) hydroxyl group from Tyr residue (Table 1 in the main text and Table S3).

In the pH range of 6-8, a slight turbidity of the Shep I - Zn(II) complex solutions in the measuring cell was observed, probably due to the accumulation of positive charge resulting from the deprotonation of numerous non-binding histidyl residues.

Table S 3. Stability constants for Shep I - Zn(II) system in aqueous solution of 4 mM $HClO_4$ for each calculated complex species with the proposed coordination modes. $C_L$ = 0,5 mM; [L] : [Zn(II)] = 1 : 0,9; $I$ = 0,1 M $NaClO_4$; T = 298 K.

| **Shep I – Zn(II)** | | | | |
|---|---|---|---|---|
| Species | log $\beta$ | p$K_a$ | pH | Proposed coordination modes |
| **$[ZnH_6L]^{7+}$** | 54,09(3) | - | 5,60 | $4N_{im}$ or two different sets of $\{2N_{im}\}$ |
| **$[ZnH_5L]^{6+}$** | 48,35(2) | 5,74 | 6,06 | $4N_{im}$ (Deprotonation of non-bonding His residue with p$K_a$ 6,54) or two different sets of $\{2N_{im}\}$ |
| **$[ZnH_4L]^{5+}$** | 42,04(2) | 6,31 | 6,75 | $4N_{im}$ (Deprotonation of non-bonding His residue with p$K_a$ 6,94) or two different sets of $\{2N_{im}\}$ |
| **$[ZnH_3L]^{4+}$** | 34,92(2) | 7,12 | 7,66 | $4N_{im}$ (Deprotonation of non-bonding His residue with p$K_a$ 7,21) or two different sets of $\{2N_{im}\}$ |
| **$[ZnH_2L]^{3+}$** | 26,72(3) | 8,20 | 8,76 | $4N_{im}$ (Deprotonation of non-bonding His residue with p$K_a$ 8,03) or two different sets of $\{2N_{im}\}$ |
| **$[ZnHL]^{2+}$** | 17,27(3) | 9,45 | 9,50 | $4N_{im}$ (Deprotonation of non-bonding N-terminal $NH_2$ with p$K_a$ 9,58) or two different sets of $\{2N_{im}\}$ |
| **$[ZnL]^{+}$** | 7,59(3) | 9,68 | 10,22 | $4N_{im}$ (Deprotonation of non-bonding Tyr residue with p$K_a$ 10,38) |

Table S 4. Positive band maxima in far-UV CD spectroscopy in water solution of 4 mM $HClO_4$ with $I$ = 0,1 M $NaClO_4$ at pH 5,50.

| **Shep I** | | **Cu(II)** | | **Zn(II)** | |
|---|---|---|---|---|---|
| **λ [nm]** | **CD [mdeg]** | **λ [nm]** | **CD [mdeg]** | **λ [nm]** | **CD [mdeg]** |
| 198,6 | 1,73 | 196,2 | 3,01 | 197,2 | 2,56 |
| 228,6 | 0,41 | 222,8 | 0,39 | 228,8 | 0,27 |

REFERENCES


(1) Watly, J.; Simonovsky, E.; Barbosa, N.; Spodzieja, M.; Wieczorek, R.; Rodziewicz-Motowidlo, S.; Miller, Y.; Kozlowski, H. African Viper Poly-His Tag Peptide Fragment Efficiently Binds Metal Ions and Is Folded into an α-Helical Structure. *Inorg Chem* **2015**, *54* (16), 7692–7702. https://doi.org/10.1021/acs.inorgchem.5b01029.

(2) Watly, J.; Simonovsky, E.; Wieczorek, R.; Barbosa, N.; Miller, Y.; Kozlowski, H. Insight into the Coordination and the Binding Sites of $Cu^{2+}$ by the Histidyl-6-Tag Using Experimental and Computational Tools. *Inorg Chem* **2014**, *53* (13), 6675–6683. https://doi.org/10.1021/ic500387u.
